# Insulin signaling pathway related m^6^A methylated biomarker for type 2 diabetes and the potential modulation mechanism

**DOI:** 10.1186/s12986-026-01086-4

**Published:** 2026-01-31

**Authors:** Jing Dong, Yu Zhang, Yan-Ling Li, Li-Juan Wu, Shuo Wang, Ning Chen, Yu-Xiang Yan

**Affiliations:** 1 Health Management Center, Xuanwu Hospital, Capital Medical University, Beijing, China; 2 Department of Epidemiology and Biostatistics, School of Public Health, Capital Medical University, No.10 Xitoutiao, YouAnMen, Beijing, 100069 P. R. China

**Keywords:** Type 2 diabetes, N6-methyladenosine, Insulin signaling pathway, Biomarker

## Abstract

**Background:**

Deficiency of insulin signaling components may act as the underlying mechanisms for insulin resistance and type 2 diabetes (T2D). N6-methyladenosine (m^6^A) is emerging as an important regulatory mechanism in gene expression at the post-transcriptional level. This study aimed to identify the insulin signaling pathway related m^6^A methylated biomarker for early detection of T2D.

**Methods:**

Candidate genes (PIK3CA and AKT1) with abnormal m^6^A modification in insulin signaling pathway and the potential methylase (FTO) were selected and validated. Luciferase assay was used to investigate the interaction between FTO and PIK3CA/AKT1. The mechanism of FTO on m^6^A modification of PIK3CA was validated by methylated RNA immunoprecipitation (MeRIP), western-blot and glucose metabolism assays. The clinical significance of m^6^A methylated PIK3CA was evaluated in a nested case-control study.

**Results:**

We found that the m^6^A content and mRNA expression of PIK3CA were significantly downregulated and FTO mRNA expression was significantly upregulated in T2D cases and prediabetes, compared with controls. Mechanism analysis demonstrated that FTO overexpression significantly reduced the m^6^A level, total mRNA and protein expression of PIK3CA, subsequently inhibited glucose consumption and glucose metabolism. m^6^A methylated PIK3CA can significantly improve the predictive ability of T2D occurrence beyond traditional risk factors alone.

**Conclusion:**

Decreased m^6^A level of PIK3CA promote abnormal glucose metabolism and T2D development by reducing PIK3CA mRNA expression, which was under the control of FTO-mediated m^6^A modification. m^6^A methylated PIK3CA is a valuable biomarker for prediction and early detection of T2D.

**Supplementary Information:**

The online version contains supplementary material available at 10.1186/s12986-026-01086-4.

## Introduction

Type 2 diabetes (T2D) has become a major public health problem worldwide owing to its high prevalence and related disability and mortality. Especially, the incidence and prevalence of T2D in young people (aged 40 years or younger) keeps on rising globally [1]. It is urgent to develop effective measures to identify the population at high risk and interventions to prevent and delay the onset of T2D. As the earliest defects in the pathogenesis of T2D, insulin resistance refers to a decrease in a target cell’s metabolic response to insulin, or an impaired lowering effect of circulating insulin on blood glucose at the whole-organism level [2]. Cohort studies have demonstrated that insulin resistance presents a long time before diagnosis, and may go on to develop pre-diabetes or T2D if timely intervention and effective lifestyle changes are not made [3].

Insulin mediates its biological effects primarily through the insulin receptor substrate (IRS) - phosphoinositide3-kinase (PI3K) - proteinkinase-B (Akt) pathway. Phosphorylation of Akt promotes translation of glucose transporter 4 (GLUT4) and ultimately stimulates the glucose transport across cell membranes. Insulin signaling pathway plays an important role in the metabolism including glucose uptake and the synthesis of glycogen and fatty acids [4, 5]. A deficiency of insulin signaling components may act as the underlying mechanisms for insulin resistance and T2D [6]. At population level, gene expression of PI3K and AKT in peripheral blood significantly downregulated in newly diagnosed T2D cases and prediabetes individuals [7]. Compared with normal controls, mRNA levels of GLUT4 in adipose tissues of the T2D cases are significantly decreased [8]. Given the importance of insulin signaling pathway, identifying new molecules that regulate the pathway may be a useful method for early detection and intervention for individuals at high risk of T2D.

As the most common internal chemical modification for mRNAs, N6-methyladenosine (m^6^A) is emerging as an important regulatory mechanism in gene expression at the post-transcriptional level and participates in various biological processes. m^6^A plays important roles in the structure, stability, processing and translation of RNA, as well as other RNA metabolic processes [9]. In the dynamic regulation of m^6^A modification, m^6^A methyltransferases and demethylases play crucial roles. Disruption of m^6^A homeostasis may led to defects in target molecular pathway and biological functions, and contribute to the development and progression of a variety of human diseases [10].

Recent studies have shown that m^6^A modification may play an important role in the development and progression of T2D, such as regulating glucose, pancreatic β-cell function, and insulin sensitivity. Moreover, m^6^A modification is associated with risk factors of T2D including obesity and dyslipidemia [11]. Previous studies demonstrated that the content of m^6^A is significantly reduced in T2D patients and m^6^A content in RNA is correlated with FTO mRNA expression [12, 13]. m^6^A-sequencing in human T2D islets reveals several hypomethylated transcripts involved in cell-cycle progression, insulin secretion, and the Insulin/IGF1-AKT-PDX1 pathway [14]. In addition, METTL14 is essential for β-survival, differentiation and insulin secretion [15]. METTL3 inhibits hepatic insulin sensitivity via m^6^A modification of FASN mRNA and promoting fatty acid metabolism, which eventually results in the development of T2D [16].

In this study, based on the Kyoto Encyclopedia of Genes and Genomes (KEGG) pathway analysis of epitranscriptome profile of T2D cases in adipose tissue, the abnormal m^6^A modification of insulin signal components, PIK3CA and AKT1, was discovered and further validated in peripheral blood. The potential role as novel biomarkers for early detection of T2D were assessed, and the potential mechanism of m^6^A modification was also explored.

## Materials and methods

### Study design and subjects

The present study was performed based on a multi-stage design. Firstly, epitranscriptome profiles were compared between four new T2D cases and four age and sex-matched controls in adipose tissue. Candidate genes (PIK3CA and AKT1) with abnormal m^6^A modification in insulin signal pathway and the potential methylase (FTO) were then selected and validated in peripheral blood using in a cohort with new T2D cases, prediabetes and health controls (70 participants per group). Secondly, luciferase assay was used to investigate the interaction between FTO and PIK3CA/AKT1. Thirdly, the mechanism of FTO on m^6^A modification of PIK3CA was validated by methylated RNA immunoprecipitation (MeRIP), western-blot, and glucose metabolism assays. Fourthly, a nested case-control study, prospectively collecting blood samples from 100 T2D cases and 100 matched controls six-year before clinical diagnosis, was performed to further assess the predictive ability of m^6^A methylated PIK3CA for T2D development.

In the first stage, all the T2D cases were aged from 30 to 65 years and were randomly selected from newly diagnosed cases during September 2020 to December 2021 in a functional community cohort [17]. The prediabetes and healthy participants in validation study were frequency matched with T2D cases according to gender and age (± 3 years). In the nested case-control study, cases were newly diagnosed T2D patients in the same cohort during 2022. Controls were selected from those who did not develop T2D during the follow-up period. For each case, one control was matched with the criteria of gender and age. The m^6^A modification levels of candidate genes at baseline in 2016 between the two groups was compared. The average time between blood collection and date of diagnosis was 6 years. We excluded those with a past history of using antidiabetic treatment or any endocrine disease other than T2D. other exclusion criteria includes: (1) liver and kidney dysfunction, (2) clinically acute or chronic inflammatory diseases, (3) severe heart diseases, (4) gastrointestinal diseases.

In accordance with the 2011 World Health Organization (WHO) criteria, T2D was defined as: (1) fasting plasma glucose (FPG) ≥ 7.0 mmol/l; (2) 2-h plasma glucose in oral glucose tolerance test (OGTT) ≥ 11.1mmol/L; (3) HbAlc > 6.5%. Prediabetes was defined as: (1) FPG ≥6.1 mmol/l and < 7.0 mmol/l; (2) 2-h plasma glucose in OGTT ≥7.8 mmol/l and < 11.1 mmol/l; (3) HbAlc 5.7–6.4%.

This study was approved by the university ethical committee and written informed consent was obtained from each participant before the investigation.

### Data collection and clinical measurements

Data collection and clinical measurements were performed during follow-up of the cohort. A structured questionnaire was used to collect information including demographic data, health-related behaviors, medication use, personal and family medical history. Physical measurements, including weight, height, waist circumference (WC) and blood pressure, were conducted by trained health workers using standard measurement. Blood biochemical parameters, including FPG, HbAlc, fasting triglyceride (TG), total cholesterol (TC), high-density lipoprotein-cholesterol (HDLC), and low-density lipoprotein-cholesterol (LDLC) were tested through standard procedures in the clinical laboratory of the hospital. Concentration of fasting insulin was determined using radioimmunoassay [7]. Homeostasis model assessment index (HOMA-IR) was calculated as: fasting insulin (µIU/mL) × fasting glucose (mmol/L)/22.5 [18].

### Sample collection and total RNA extraction

Overnight fasting venous blood samples were collected from each participant. Peripheral blood mononuclear cells (PBMCs) were isolated using density gradient centrifugation. Human adipose tissues were obtained from Department of Neurosurgery, Xuanwu Hospital, Capital Medical University. Total RNA was extracted continuously using the TRIzol reagent (Invitrogen, USA) according to the manufacturer’s instructions. The purity and amount of RNA were determined using NanoDrop ND-1000. The average A260/A280 was 1.8 to 2.0 and 28 S/18S was > 1.9.

### Transcriptomic microarray analysis

The transcriptomic microarray assay was performed using Arraystar Human mRNA&lncRNA Epitranscriptomic Microarray (8 × 60 K) according to the manufacturer’s protocol. Briefly, the total RNAs were immunoprecipitated with m^6^A antibody. The modified RNAs were eluted from the immunoprecipitated magnetic beads as the “IP”. The unmodified RNAs were recovered from the supernatant as “Sup”. The “IP” and “Sup” RNAs were labeled with Cy5 and Cy3 respectively as cRNAs in separate reactions using Arraystar RNA Labeling kit. The cRNAs were mixed and hybridized onto microarray. The arrays were then washed and scanned using an Agilent scanner G2505C. Data were extracted using Feature Extraction (11.0.1.1). Raw intensities of Cy5-labeled RNA and Cy3-labeled RNA were normalized with average of log2-scaled Spike-in RNA intensities. The m^6^A methylation level was calculated for the percentage of modification based on the Cy5-labeled and Cy3-labeled normalized intensities. Differentially m^6^A-methylated and differentially expressed genes were screened using the “limma” package in R program. To address the issue of multiple comparisons, the Benjamini-Hochberg procedure to control the False Discovery Rate (FDR) was applied. We used threshold values of fold change (FC) > 2 or < 2 with FDR < 0.05. KEGG pathway analysis was performed to reveal the potential functions of genes with significant differences in both gene expression and m^6^A modification using the “clusterProfiler” package.

Genes with significant differences in both gene expression and m^6^A modification, and were involved in the insulin signaling pathway were identified as candidate genes.

### Methylated RNA immunoprecipitation (MeRIP)

MeRIP m^6^A kits (Millipore Corp.) were applied for evaluation of the m^6^A level of candidate genes. According to the instructions of the kit, chemically fragmented RNA was incubated with m^6^A antibody for immunoprecipitation. Enrichment of m^6^A containing mRNA was then analyzed by quantitative reverse-transcription polymerase chain reaction (RT-qPCR) normalized with the input RNA, and each experiment was repeated three times independently. Samples from different experimental groups were randomized on the assay plates to avoid confounding group differences with plate position effects.

### Real-time quantitative PCR

RT-qPCR was used to confirm the mRNA expression of candidate genes. First-strand cDNA was synthesized from total RNA (1 µg) using the reverse transcription kit PrimeScript™ RT Master Mix (Takara, Japan). The RT-qPCR reactions were performed using 2 µl of the first strand cDNA with SYBR Premix Ex TaqTM kit (Takara) in a 20-µl reaction volume on Bio-Rad iQ5 Real Time PCR System (Bio-Rad, USA). The amplification conditions were 95 °C for 10 min, followed by 45 cycles of 95 °C for 15 s and 60 °C for 60 s. GAPDH was used as an internal reference and all reactions were performed in triplicate. Data analysis was performed by the 2^−ΔΔCt^ method.

### Dual-luciferase reporter assays

The DNA fragments of the PIK3CA-untranslated regions (3’UTRs) containing the wild-type (WT) m^6^A motifs, as well as mutant (MUT) motifs, were synthesized by GeneChem (Shanghai, China). PIK3CA-WT and PIK3CA-MUT were inserted into downstream of firefly luciferase in a GV272 vector. For the luciferase reporter assay, PIK3CA-WT or PIK3CA-MUT and FTO-OE (overexpression) or FTO-NC (negative control) plasmids were cotransfected into 293T cells in 6-well plates. Similarly, AKT1-WT or AKT1-MUT and FTO-OE or FTO-NC plasmids were cotransfected into 293T cells. The relative luciferase activities were assessed 48 h after transfection by the Dual-Luciferase Reporter Assay System (Promega, Madison, USA). Each group was repeated in triplicate.

### Cell transfection

HepG2 cells were cultured in Dulbecco’s modified Eagle’s medium (DMEM) containing 10% fetal bovine serum (FBS). The small interfering RNA (siRNA) targeting FTO and scrambled (control) siRNA were purchased from Genechem Co., Ltd. (Shanghai, China). A FTO overexpression (OE) vector containing the coding sequence was constructed using PCR-generated fragments cloned into the lentiviral vector, GV492. All constructs were confirmed by DNA sequence analysis. An empty vector was used as the negative control (GV492-NC). During the course of transfection, the FTO knock-down (KD), FTO-OE vectors and their controls were transfected into HepG2 cells (1 × 10^6^), respectively, using Lipofectamine 3000 (Invitrogen) according to the manufacturer’s instructions. The western blot assay was used to evaluate the level of FTO protein expression after transfection. The scrambled (control) siRNA and an empty vector were used as controls, respectively.

### Western blot

The proteins were separated and detected using an automated capillary electrophoresis system (Simple Western System and Compass software; Protein Simple, USA). The ProteinSimple kit included a biotinylated ladder, antibody diluent, streptavidin-horseradish peroxidase (HRP), anti-rabbit secondary antibody, luminol-peroxide mix, and wash buffer. After preparing the samples, the kit was added to matching plate wells according to the manufacturer’s instructions. Antibodies against the following proteins were used (Abcam, Cambridge, UK): FTO (ab124892, 1:1000), β-actin (ab124964, 1:1000), PIK3CA (ab191606, 1:1000) and phospho-PIK3CA (ab182651). The antibody dilutions were: 1:1,000. Signals were detected using an HRP-conjugated secondary anti-rabbit antibody and visualized using ProteinSimple software.

### Glucose consumption and glycogen content assays

Glucose consumption from the culture media was determined using a glucose assay kit. Briefly, after transfection, HepG2 cells were treated with insulin (100 nM) for 30 min. The cells were then washed with PBS twice and the medium was replaced by RPMI-1640 containing 11.1 mmol/L glucose supplemented with 0.2% BSA. The supernatant was then collected and glucose consumption was calculated by the glucose concentration of blank well minus glucose concentrations of the well with cells by using the glucose kit (Nanjing Jiancheng, China). Glucose consumption was detected at 6, 12, 24 and 36 h. The CCK8 assay was used to adjust for the glucose consumption. Experiments were performed in six replicates.

Cells were then harvested and lysed in lysis buffer. The cellular glycogen content was evaluated using a glycogen content detection kit (BC0340; Solarbio) with spectrophotometer at a wavelength of 620 nm and normalized with protein level. Experiments were performed in three replicates.

### Statistical analysis

Data were represented as the mean ± standard deviation (SD) or percentages when they fit. Differences between the study groups were analyzed using the chi-square test, Student’s t-test, or one-way analysis of variance (ANOVA). Spearman’s correlation coefficients were used to evaluate the relationship between variables. Multivariate logistic regression was performed to investigate the association between m^6^A level of PIK3CA (grouped by median since the data shows a skewed distribution with extreme outliers) and the risk of T2D after adjusting for confounders that may be related to T2D, such as smoking, drinking, physical activity, BMI, SBP, FPG, TG and LDLC. Receiver operating characteristic (ROC) curve was constructed to assess the predictive ability for T2D. Net reclassification improvement (NRI) and integrated discrimination improvement (IDI) were used to estimate the improvement of predictive ability after adding baseline m^6^A methylated PIK3CA to traditional model. Statistical analyses were conducted using SPSS 26.0, GraphPad Prism 5.01 and R 4.4.1. A two-sided *P* < 0.05 was considered statistically significant.

## Results

### Microarray analysis

**Fig. 1:**
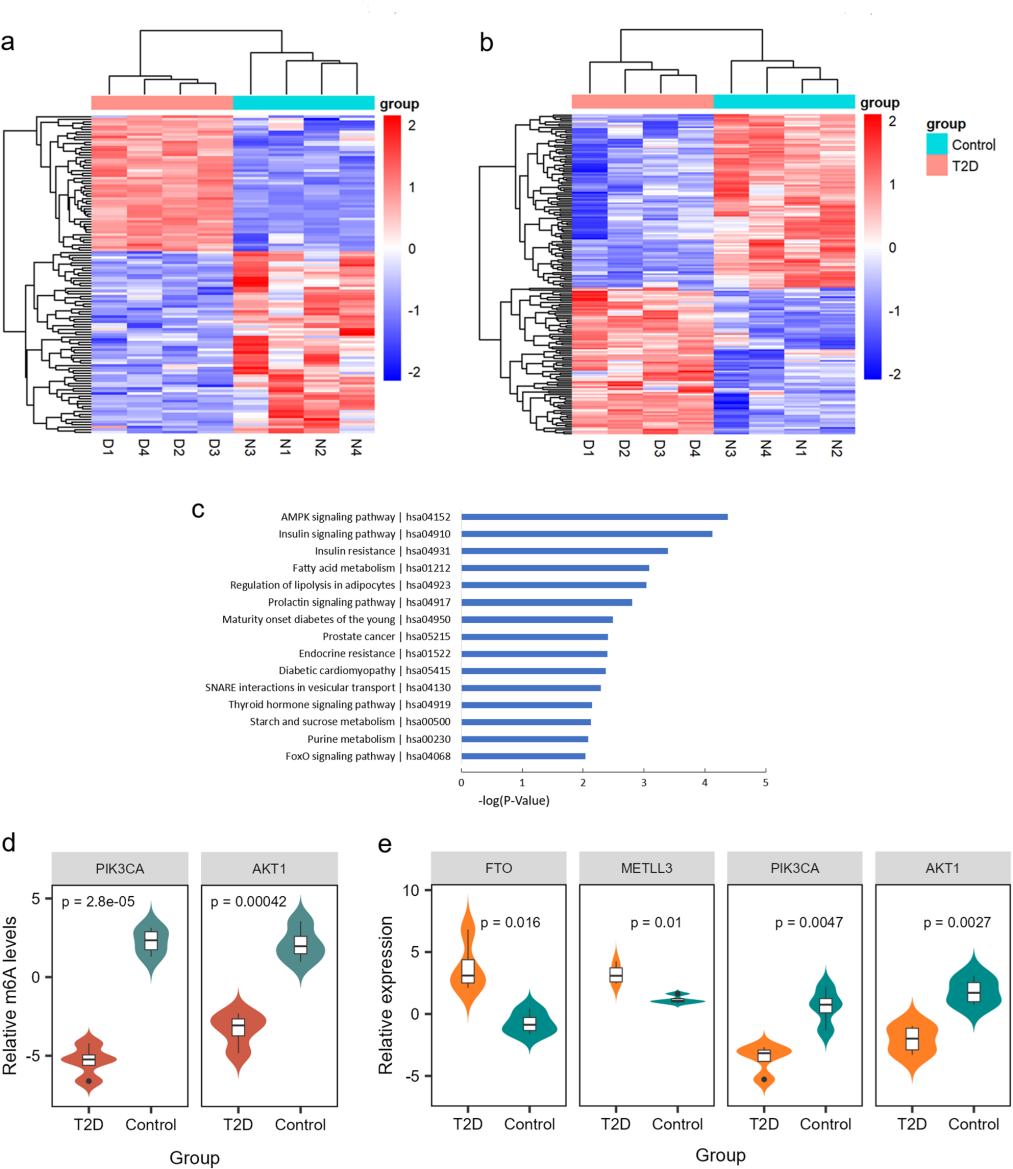
Differential m^6^A modification and expression profiles between type 2 diabetes (T2D) and controls in microarray under the cut‑off criteria |log2FC| >1 and FDR < 0.05. (**a**) Heat map of hierarchical cluster analysis for differentially m^6^A-methylated genes between T2D cases and controls. Red and blue denote high and low expression, respectively. (**b**) Heat map of hierarchical cluster analysis for differentially expressed genes between T2D cases and controls. (**c**) Significantly highly enriched KEGG pathways of genes with significant differences in both m^6^A modification and mRNA expression. (**d**) Comparation of m^6^A levels of candidate genes between T2D cases and controls. (**e**) Comparation of expression levels of candidate genes

Using a threshold of |log2FC| > 1 and FDR < 0.05, microarray analysis identified 124 differentially m^6^A-methylated genes (43 hyper- and 71 hypo-methylated) and 181 differentially expressed genes (83 upregulated and 98 downregulated) between four T2D cases and four controls (Supplementary Table 1). Hierarchical clustering of both m^6^A modification and expression profiles correctly classified the two groups (Fig. 1a-b). Among these, 60 genes exhibited concurrent differential methylation and expression. The top 15 significantly enriched KEGG pathways annotated by these genes, including AMPK signaling pathway, insulin resistance, fatty acid metabolism, regulation of lipolysis in adipocyte s and endocrine resistance, et al. (Fig. 1c). The insulin signaling pathway ranked second among the enriched pathways, indicating that m^6^A modification may regulate insulin signaling-related genes. Consistent with this, both m^6^A modification and expression of PIK3CA and AKT1 were significantly reduced in T2D group, whereas the RNA methylase METTL3 and demethylase FTO were upregulated (Fig. 1d-e). Since FTO is an important demethylase, we infer that down-regulation of PIK3CA and AKT1 were caused by decreased m^6^A modification that were regulated by FTO.

### MeRIP-qPCR and RT-qPCR validation for candidate genes

**Fig. 2:**
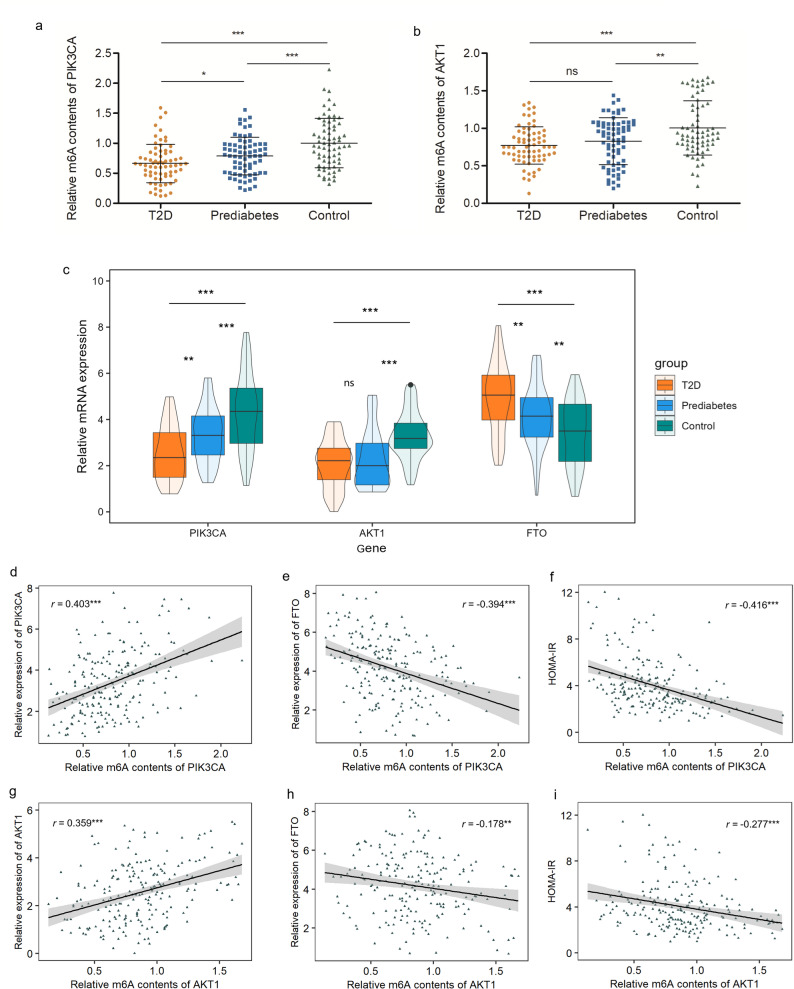
Validation of m^6^A level and mRNA expression of candidate genes in a large cohort with 70 T2D cases, 70 prediabetes and 70 controls. (**a**) Comparation of m^6^A levels of PIK3CA between the groups. (**b**) Comparation of m^6^A levels of AKT1 between the groups. (**c**) Comparation of mRNA expression of PIK3CA, AKT1 and FTO between the groups. (**d**-**f**) Spearman’s correlation analysis between PIK3CA m^6^A content with PIK3CA mRNA, AKT1 mRNA and HOMA-IR. (**h**-**i**) Spearman’s correlation analysis between PIK3CA m^6^A content with PIK3CA mRNA, AKT1 mRNA and HOMA-IR. ^*^ *P* < 0.05, ^**^ *P* < 0.01, ^***^ *P* < 0.001

To validate the microarray data, MeRIP-qPCR was performed to compare the m^6^A methylated levels of PIK3CA and AKT1 in PBMCs between new T2D cases, prediabetes and health controls (Table 1). RT-qPCR was performed to compare the mRNA expression of PIK3CA, AKT1 and FTO in PBMCs between the groups. Primer sequences are listed in Supplementary Table 2. We found that the m^6^A contents and mRNA expression of PIK3CA and AKT1 were significantly down-regulated in T2D cases and prediabetes, compared with control group (Fig. 2a-c). In addition, the m^6^A contents and mRNA expression of PIK3CA in T2D cases were significantly lower than prediabetes groups. In contrast, the mRNA expression of FTO were significantly increased in T2D cases and prediabetes (Fig. 2c). Spearman’s correlation analysis showed that m^6^A contents of PIK3CA are significantly positively correlated with mRNA of PIK3CA, and negatively correlated with FTO expression and HOMA-IR (Fig. 2d-f). The correlation also existed when data were stratified according to sub-groups (Supplementary Table 3). Although similar association existed between m^6^A contents of AKT1 and AKT1/FTO expression and HOMA-IR in all the subjects, the association were not significant in each sub-group (Fig. 2g-i and Supplementary Table 4).

**Table 1:** Demographic and clinical characteristics of study participants in the validation study

Variable	T2D (*n* = 70)	Prediabetes (*n* = 70)	Control (*n* = 70)	*P*
Age (years)	51.97 ± 8.85	51.83 ± 9.64	51.71 ± 10.06	0.600^#^
Gender (male/female)	39/31	40/30	37/33	0.874^*^
BMI (kg/m^2^)	25.85 ± 2.95^a^	25.56 ± 2.96^a^	23.91 ± 2.68	< 0.001^#^
WC (cm)	86.69 ± 8.69^a^	85.65 ± 9.27^a^	80.40 ± 9.36	< 0.001^#^
SBP (mmHg)	132.02 ± 10.21^a^	129.56 ± 9.30^a^	123.43 ± 13.61	< 0.001^#^
DBP (mmHg)	82.56 ± 8.72^a, b^	79.07 ± 8.95^a^	75.80 ± 10.36	< 0.001^#^
TC (mmol/l)	4.94 ± 1.08	4.85 ± 1.07	4.64 ± 0.72	0.160^#^
TG (mmol/l)	2.23 ± 1.71	1.75 ± 1.01	1.42 ± 1.21	0.001^#^
LDLC (mmol/l)	3.13 ± 1.14	3.06 ± 1.01	2.74 ± 0.87	0.014^#^
HDLC (mmol/l)	1.47 ± 0.39	1.43 ± 0.39	1.58 ± 0.43	0.105^#^
FPG (mmol/l)	9.22 ± 2.28^a, b^	6.52 ± 0.26^a^	5.13 ± 0.47	< 0.001^#^
HbA1c (%)	7.71 ± 1.36^a, b^	5.77 ± 0.41^a^	5.16 ± 0.40	< 0.001^#^
Insulin (uIU/ml)	15.28 ± 2.88^a, b^	12.58 ± 2.30^a^	9.85 ± 2.15	< 0.001^#^
HOMA-IR	6.35 ± 2.37^a, b^	3.63 ± 0.72^a^	2.24 ± 0.60	< 0.001^#^
Smoking (n, %)	8, 11.43	8, 11.43	6, 8.57	0.816^*^
Alcohol use (n, %)	10, 14.29	9, 12.86	7, 10.00	0.735^*^
Physical activity (n, %)	58, 82.86	60, 85.71	62, 88.57	0.627^*^

BMI: body mass index, WC: waist circumference, SBP: systolic blood pressure, DBP: diastolic blood pressure, TC: total cholesterol, TG: triglyceride, LDLC: low-density lipoprotein cholesterol, HDLC: high-density lipoprotein cholesterol, FPG: fast plasma glucose, HOMA-IR: homeostasis model assessment of insulin

^#^ One-way ANOVA; ^*^ Chi-square test

^a^ Significantly different from control group (*P* < 0.05)

^b^ Significantly different from prediabetes group (*P* < 0.05)

### Interaction of FTO and PIK3CA/AKT1

**Fig. 3:**
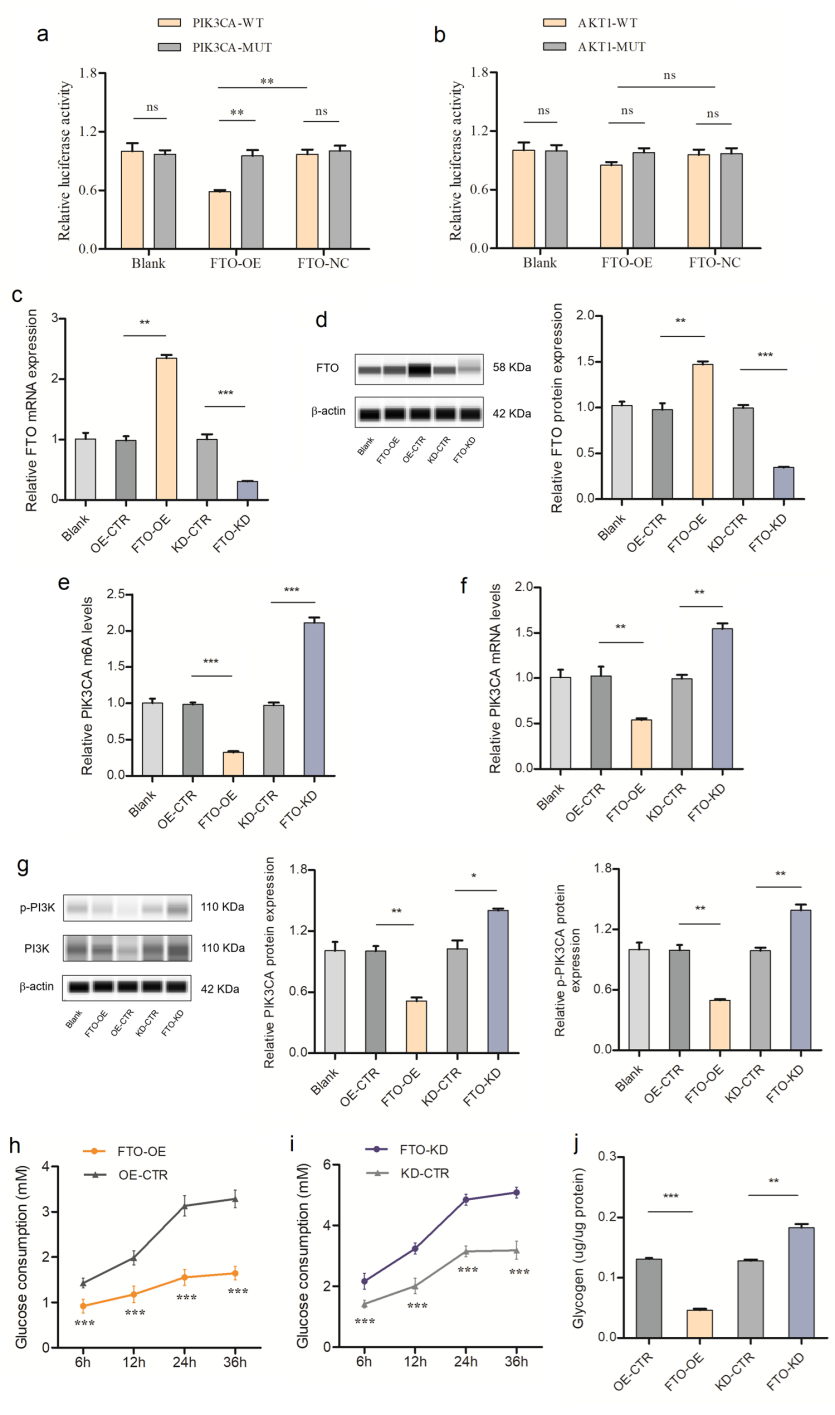
Validation of PIK3CA being subjected to FTO-mediated m^6^A modification and involved in glucose metabolism. (**a**) Luciferase assays revealed that modulation of PIK3CA expression was under the control of FTO. (**b**) Luciferase assays revealed that modulation of AKT1 expression was not under the control of FTO. (**c**) Relative mRNA expression of FTO. (**d**) Relative protein expression of FTO. (**e**) Relative m^6^A level of PIK3CA. (**f**) Relative mRNA expression of PIK3CA. (**g**) Relative protein expression of PI3K and phospho-PI3K (p- PI3K). (**h**-**j**) Glucose consumption and glycogen content in HepG2 cells. ^*^ *P* < 0.05, ^**^ *P* < 0.01, ^***^ *P* < 0.001

The dual-luciferase reporter assays were performed to investigate whether FTO regulated the expression of PIK3CA/AKT1 via recognition of their mRNA m^6^A motifs in the 3’-UTR. The sequences of the wild-type and mutant cDNA fragments are listed in Supplementary Table 5. Compared to the co-transfection PIK3CA-MUT group, the relative luciferase activity was significantly lower in the FTO-OE plasmid and PIK3CA-WT co-transfection group, indicating that there was a direct interaction between FTO and PIK3CA. In contrast, overexpression of FTO showed no effect on the expression of the mutant PIK3CA-fused reporter, suggesting the modulation of PIK3CA expression was under the control of FTO-associated m^6^A modification. Meanwhile, transfection with FTO-OE did not significantly reduce the activity of the luciferase reporter carrying AKT1-WT compared with that of the AKT1-MUT (Fig. 3a-b).

### Regulatory effect of FTO on PIK3CA

To further address the effect of m^6^A modification on PIK3CA expression, we constructed both FTO knockdown and overexpression vectors. The transfection of HepG2 cells with overexpression vectors significantly increased FTO mRNA and protein expression. In contrast, co-transfection with knockdown vectors significantly decreased FTO mRNA and protein expression in HepG2 cells (Fig. 3c-d).

As expected, overexpression of FTO dramatically reduced the m^6^A level of PIK3CA mRNA and downregulated mRNA expression of PIK3CA, compared with the control group. On the contrary, we found that knockdown of FTO substantially augmented the m^6^A level of PIK3CA mRNA and up-regulated mRNA expression of PIK3CA (Fig. 3e-f). Together, our findings suggest that FTO-mediated m^6^A modification repressed PIK3CA expression through demethylating m6A in RNA in vivo.

Western blot analysis further revealed a significant decrease in PIK3CA protein expression and phosphorylation of PIK3CA in HepG2 cells after overexpression of FTO compared with the control group, indicating that the insulin signaling pathway was suppressed. However, significant higher levels of PIK3CA expression and phosphorylation of PIK3CA were observed after knockdown of FTO (Fig. 3g).

### Regulatory effect of FTO on glucose metabolism

Furthermore, we found that glucose consumption and glycogen content in the FTO over-expression group were significantly reduced compared with the control group. In contrast, suppression of FTO can significantly improve glucose consumption in medium and promote glycogen synthesis in the HepG2 cells (Fig. 3h-i). These findings indicate that FTO regulate glucose metabolism by mediating m^6^A modification of PIK3CA and affect the activation of insulin signaling pathway.

### Association between and m^6^A levels of PIK3CA and risk of T2D

**Fig. 4:**
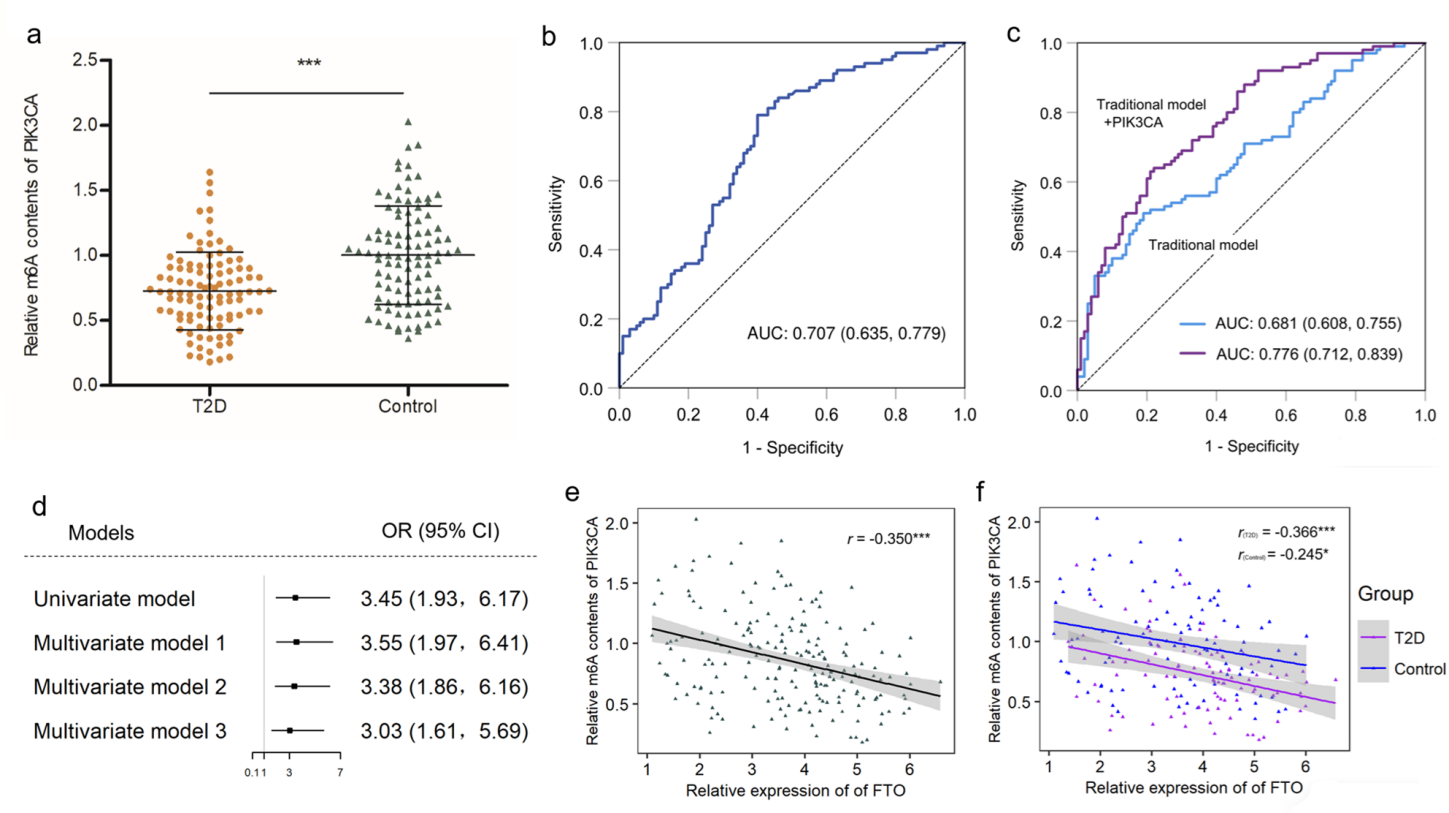
Evaluation of the clinical significance of m^6^A-methylated PIK3CA as a potential biomarker for T2D. (**a**) Comparation of baseline m^6^A levels of PIK3CA among 100 T2D cases and 100 matched controls. (**b**-**c**) ROC plots of m^6^A-methylated PIK3CA as a predictor of T2D. (**d**) Univariate and multiple logistic regression analysis for the risk of T2D (high m^6^A level was defined as reference). 1 Adjusted for smoking, drinking and physical activity. 2 Further adjusted for BMI based on model (1) 3 Further adjusted for SBP, DBP, FPG, TG and LDLC based on model (2) (**e**-**f**) Spearman’s correlation analysis between m^6^A level of PIK3CA and FTO expression. CI: confidence interval, T2D: type 2 diabetes, OR: odds ratio. ^*^ *P* < 0.05, ^***^P < 0.001

In the fourth part of the study (nested case-control study), the demographic and clinical characteristics of the newly diagnosed T2D cases and their matched controls at 6 years prior to the diagnosis (baseline) were presented in Supplementary Table S6. The relative m^6^A contents of PIK3CA in the T2D cases was significantly lower than the controls (*P* < 0.001), and could effectively predict the risk of T2D (Fig. 4a-b). Compared with the traditional factor predictive model (including BMI, SBP, TG, LDLC and FPG), the AUC of ROC curve significantly increased (from 0.681 [95%: 0.608–0.755] to 0.776 [95%CI: 0.712–0.839], *P* = 0.002) with adding PIK3CA as a predictor (Fig. 4c). With inclusion of baseline m^6^A contents of PIK3CA to the predictive model, significant reclassification improvement (NRI = 0.52, 95%CI: 0.208–0.813) and integrated discrimination improvement (IDI = 0.098, 95%CI: 0.058–0.138, *P* < 0.001) were also observed, which indicates that m^6^A methylated PIK3CA can significantly improve the predictive ability of T2D occurrence beyond traditional risk factors alone.

When m^6^A contents of PIK3CA were grouped according to the median (0.8225), participants with lower m^6^A level had a 3.03-fold (95% CI: 1.61–5.69) increased risk for T2D, compared with those with higher m^6^A level (Fig. 4d). Meanwhile, we found that the baseline mRNA expression of FTO in the T2D cases was significantly upregulated compared with the controls (3.93±1.17 vs. 3.38±1.28, *P* = 0.002). Significant negative correlation between m^6^A content of PIK3CA and FTO expression was also observed at baseline, especially in the T2D group (Fig.

## Discussion

In the present study, we identify a novel regulatory mechanism in which FTO-mediated m6A demethylation directly suppresses PIK3CA expression, leading to impaired glucose metabolism. Importantly, we establish circulating m^6^A-methylated PIK3CA as a novel biomarker for T2D development.

These findings provide a new epitranscriptomic perspective on insulin signaling dysfunction in T2D. PIK3CA encodes the p110α catalytic subunit of PI3K, a central node in the insulin signaling pathway whose altered activity is implicated in involve in the pathogenesis of several diseases including T2D [19, 20, 21]. While decreased PIK3CA expression in T2D has been previously reported [7], our study reveals that this down-regulation is driven, at least in part, by FTO-dependent m^6^A RNA demethylation. This mechanism directly links mRNA modification to the disruption of a key insulin-signaling component, offering a new insight into the molecular basis of insulin resistance.

The FTO gene is expressed in multiple tissues relevant to metabolic diseases, including adipose tissue, skeletal muscle and liver. Its expression and protein levels have been positively correlated with obesity, insulin resistance, hyperglycemia, and diabetic complications [22, 23]. Recently, FTO was identified as a powerful m^6^A demethylases can effectively demethylate m^6^Am and m^6^A [24]. Consistent with this, prior clinical observations report decreased global m^6^A levels in white blood cells of T2D patients, negatively correlating with FTO mRNA [12]. In our cohort, we also found significantly elevated FTO expression and concurrently reduced m^6^A content of PIK3CA in both T2D and prediabetes groups compared to controls. Notably, this alteration was already present at baseline in individuals who later developed T2D, as shown in our nested case-control study. The consistent negative correlation between FTO expression and PIK3CA m^6^A levels across all subjects and subgroups strongly suggests that FTO actively reduces m^6^A methylation on PIK3CA transcripts. Cellular and molecular experiments further confirmed PIK3CA as a direct downstream target of FTO-mediated m^6^A demethylation. Functionally, overexpression of FTO and subsequent reduction of PIK3CA m^6^A further inhibit cellular glucose uptake and glycogen synthesis, while FTO knockout improves glucose metabolism by restoring PIK3CA m^6^A levels. For these mechanistic assays, we utilized HepG2 and 293T cell models. HepG2 cells, derived from liver, are a standard model for studying glucose metabolism and insulin signaling pathways, while 293T cells offer high transfection efficiency and are widely used for luciferase reporter and protein interaction studies.

The biological implications of the observed FTO–PIK3CA–HOMA-IR relationship are profound. Insulin resistance is a core pathophysiological defect in T2D, often emerging years before clinical diagnosis [3]. Our data delineate a coherent mechanistic chain: elevated FTO drives m^6^A demethylation of PIK3CA mRNA, leading to its downregulation; reduced PIK3CA expression then impairs PI3K activity, contributing to insulin resistance, as reflected by the positive correlation between PIK3CA m^6^A content and its mRNA level, and the inverse correlation with HOMA-IR. Our findings reveal an epitranscriptomic pathway connects key insulin signaling defect with reversible RNA modification.

The predictive efficiency of m^6^A methylated PIK3CA for T2D was investigated in a nested case-control study, which allowed for an estimation of its causal relationship with development of T2D. The measurement of m^6^A content of PIK3CA and other potential risk factors were prior to the diagnosis of T2D. The decreased PIK3CA m^6^A mRNA at baseline were associated with increased T2D risk, and remained an independent predictor of T2D even after adjustment for covariates. By incorporating baseline m^6^A content of PIK3CA into the traditional model (including BMI, SBP, TG, LDLC and FPG), the predictive ability of new model for T2D occurrence was significantly improved (NRI > 0, *P*_IDI_ <0.05).

These findings offer promising translational applications. First, reduced circulating m^6^A methylated PIK3CA may serve as an early biomarker for T2D risk, enabling screening in prediabetes. Second, its quantification could refine risk stratification among individuals with similar conventional profiles. Third, tracking this marker may dynamically reflect metabolic status or intervention response. Ultimately, targeting this axis by inhibiting FTO or stabilizing m^6^A modifications could restore insulin signaling in T2D. Previous studies demonstrated that total m^6^A in peripheral blood RNA was highly effective for the detection of T2D and m^6^A landscape segregates human T2D from controls significantly better than the transcriptome [13, 14]. With the rapid development of biotechnology, the application of m^6^A in disease treatment, intervention, and high-risk population screening holds significant potential.

Collectively, our findings suggest that decreased m^6^A level of PIK3CA promote abnormal glucose metabolism and T2D development by reducing PIK3CA mRNA expression, which was under the control of FTO-mediated m^6^A modification. m^6^A methylated PIK3CA is a valuable biomarker for prediction and early detection of T2D. Our findings add a new layer of epigenetic alterations that contribute to the development of T2D and unveil a novel target for the prevention and treatment of T2D. The results are reliable based on a multistage design. However, several limitations of this study should be acknowledged.

First, although the statistical power of both validation study (0.925) and case-control study are reasonable (0.959), the overall sample size, particularly for some stratified analyses, remains relatively modest. Second, the investigation utilized tissues from different sources (adipose and PBMCs). While both are relevant to metabolism, potential heterogeneity in m6A regulation between these tissues warrants consideration. Third, although we used established cell lines (HepG2 and 293T) for mechanistic validation, which offer technical advantages, these models do not fully represent the primary metabolic tissues (e.g., adipocytes, skeletal muscle) most relevant to T2D pathophysiology. Future studies using adipocyte or myocyte models would strengthen the physiological relevance of these findings. Fourth, detailed information on participants’ dietary habits, medication use, and lifestyle factors was not available, which could influence association of m^6^A modifications and metabolic outcomes. Fifth, the promising predictive performance of circulating m^6^A methylated PIK3CA requires validation in an independent, larger prospective cohort to confirm its robustness and generalizability. Finally, further mechanistic studies are needed to clarify how m^6^A precisely regulates PIK3CA mRNA in T2D pathogenesis and to explore whether the role of m^6^A-modified PIK3CA in T2D is part of a shared pathogenic pathway across diseases or exhibits disease-specific features.

## Supplementary Information

Below is the link to the electronic supplementary material.

Supplementary Material 1

## Data Availability

The datasets used and/or analyzed during this study are available from the corresponding author on reasonable request.
