# Supplementary material for: Insulin signaling pathway related m6A methylated biomarker for type 2 diabetes and the potential modulation mechanism
Source: Nutr Metab (Lond). 2026 Jan 31;23:28. doi: 10.1186/s12986-026-01086-4 (PMC12896114; doi:10.1186/s12986-026-01086-4)
Supplement: Supplementary file 1 — Supplementary Material 1 [file 12986_2026_1086_MOESM1_ESM.docx]

Supplementary Table 1. Demographic and clinical characteristics of participants in the transcriptomic microarray study

| Variable | T2D  (n=4) | Control  (n=4) | *P* |
| --- | --- | --- | --- |
| Age (years) | 52.75 ± 5.32 | 52.75 ± 5.32 | — |
| Gender (male/female) | 2/2 | 2/2 | — |
| BMI (kg/m^2^) | 25.67 ± 2.97 | 21.80 ± 1.78 | 0.079 |
| WC (cm) | 88.25 ± 7.23 | 74.75 ± 12.15 | 0.133 |
| SBP (mmHg) | 128.50 ± 5.97 | 124.75 ± 6.40 | 0.424 |
| DBP (mmHg) | 75.75 ± 7.89 | 72.25 ± 10.63 | 0.616 |
| TC (mmol/l) | 4.56 ± 0.49 | 4.29 ± 0.23 | 0.366 |
| TG (mmol/l) | 2.15 ± 1.30 | 1.42 ± 0.61 | 0.345 |
| LDLC (mmol/l) | 3.06 ± 0.74 | 2.30 ± 0.58 | 0.154 |
| HDLC (mmol/l) | 1.32 ± 0.33 | 1.42 ± 0.35 | 0.694 |
| FPG (mmol/l) | 9.63 ± 1.42 | 4.93 ± 0.37 | 0.001 |
| HbA1c (%) | 8.20 ± 1.34 | 5.15 ± 0.21 | 0.019 |
| Smoking (n) | 0 | 0 | — |
| Alcohol use (n) | 0 | 0 | — |
| Physical activity (n) | 4 | 4 | — |

BMI: body mass index, WC: waist circumference, SBP: systolic blood pressure, DBP: diastolic blood pressure, TC: total cholesterol, TG: triglyceride, LDLC: low-density lipoprotein cholesterol, HDLC: high-density lipoprotein cholesterol, FPG: fast plasma glucose, T2D: type 2 diabetes.

Supplementary Table 2. Primers sequence for RT-qPCR

| Genes | Sequence | Sizes(bp) |
| --- | --- | --- |
| *PIK3CA* | Forward: 5'-GGTGGAATGAATGGCTGAATTATG-3'  Reverse: 5'-AGAGCAAATGGAAAGGCAAAGT-3' | 80 |
| *AKT1* | Forward: 5'-CCACGCTACTTCCTCCTCAA-3'  Reverse: 5'-CTCACGTTGGTCCACATCCT-3' | 78 |
| *FTO* | Forward: 5'-CTTCACCAAGGAGACTGCTATTTC-3'  Reverse: 5'-CAAGGTTCCTGTTGAGCACTCTG-3' | 129 |
| *METTL3* | Forward: 5'-GCAACGCATCATTCGGACAG-3'  Reverse: 5'-ACCAACCAAGCAGTGTTCCTT-3' | 70 |
| *GAPDH* | Forward: 5'-CCTCTGACTTCAACAGCGACAC-3'  Reverse: 5'-TGGTCCAGGGGTCTTACTCC-3' | 138 |

Supplementary Table 3 Spearman correlation between the m^6^A level of PIK3CA and mRNA expression of PIK3CA/FTO and HOMA-IR

|  | PIK3CA | FTO | HOMA-IR |
| --- | --- | --- | --- |
| Total subjects | 0.403^***^ | -0.394^***^ | -0.416^***^ |
| T2D group | 0.310^**^ | -0.309^**^ | -0.253^*^ |
| Prediabetes group | 0.246^*^ | -0.324^**^ | -0.294^*^ |
| Control group | 0.280^*^ | -0.308^**^ | -0.222 |

T2D: type 2 diabetes, HOMA-IR: homeostasis model assessment of insulin.

^*^ *P*<0.05, ^**^*P*<0.01, ^***^*P*<0.001.

Supplementary Table 4 Spearman correlation between the m^6^A level of AKT1 and mRNA expression of AKT1/FTO and HOMA-IR

|  | PIK3CA | FTO | HOMA-IR |
| --- | --- | --- | --- |
| Total subjects | 0.359^*^ | -0.178^**^ | -0.277^***^ |
| T2D group | -0.027 | -0.134 | -0.089 |
| Prediabetes group | 0.089 | -0.094 | -0.157 |
| Control group | 0.049 | -0.015 | -0.024 |

T2D: type 2 diabetes, HOMA-IR: homeostasis model assessment of insulin.

^*^ *P*<0.05, ^**^*P*<0.01, ^***^*P*<0.001.

Supplementary Table 5. The sequences of DNA fragments in the constructed vectors

| DNA fragments | Sequences |
| --- | --- |
| PIK3CA-3UTR-WT-plasmid | AAAGATAACTGAGAAAATGAAAGCTCACTCTGGATTCCACACTGCACTGTTAATAACTCTCAGCAGGCAAAG**A**CCGATTGCATAGGAATTGCACAATCCATGAACAGCATTAGAATTTACAGCAAGAACAGAAATAAAATACTATATAATTTAAATAATGTAAACGCAAACAGAGAATGAAAATTTCTTATTTTTCCATTGCTGTTCAATTTATAGTTTGAAGTGGGTTTTTGACTGCTTGTTTAATGAAGAAAAATGCTTGGGGTGGAAGGG**A**CTCTTGAGATTTCACCAGAGACTTTTTCTTTTTAATAAATCAAACCTTTTGATGATTTGAGGTTTTATCTGCAGTTTTGGAAGCAGTCACAAATGAGACCGAATTAACACAATTTAGACTATCTTCCTGATTCCTTAAACCCCTTTACTGAAGTATACTCATGAATAATACTTTAAAATATGGGGGAATAGAAACCATGA**A**CTTTTTACCTTTTTAAACTATTTATCCATATCTCCAAAGTAGAACATTAAACCATTTTAAGATATGTCTCATTCCCAAGTAGTCAGAGCTCACTCTCCAACTTTATTAAATACTATTTGAGCACAGG**A**CACATTCTTAAACATTTTGAAAAACATTAACCCAAGATGTAGAGGCTACTGCTAGTCGTCATTCTAGAATCTGATATTTTACTCTGTATTTGAAATGAATAGAGTGAACATTGTATACTCTAGTAAAACAGCATCACTTTAAAAATATTCATTTATGAAATCTGTTACCTATAGTTGAAGTCTTGAGTAGTGAACAAGGG**A**CTCTAATACCAATACTCTTAATATCTGGCTATTTTAGATCCCTTAAAGGGCATAATTATTGGAAATTTAGGTATTTCACTAAAGCATGTATATAATATTGCATCCCAGCCTTTTCAATATCAGGGTTAAATTATAGGAAAACTCAGTAAAATGGTACAAATCTGAAAGTTTGATGGTAGAAACTGAAGATTTAACAGAGAACTGTGTTTTACCCGAGTGCCAAAAATGCTGTGAGCCTCCTTGCACAAAATTTATACCACTTTTGCATTTTTATCTATCAGTCCAGATAGTTGTCTCCCCTGCACAAACCACATGGCCGATTTCACCATTTACATTTATTTTCAAAAGTTACTACAACCAAATTAATTCTATTAGAAGAAATGTAGACAAATTCTATAAAG**A**CTATAGATTGTGACCTAAGAAAGAAATGAGGCAAAGAACCAAACATTGAATTAAATGCTACATGGGTGACTAAGATCTGTTTCAAGTCAGTGATAATATAATTTGGGGCATGTCTTTAAGAGAAGGCTGAAAGTTGTGAGAGTATATTGTATACCGTAAGAGAATCAACTCTTCATCATGGATGGGATTGTGAAGGCTGA**A**CTATAAAATTCAGCATTGACAGCATCCTCAATTAATAATTCTTGGTGACAGAATAATACAGCTGGGCTGTTTTTTAAAATATAAACAATACCATTTTTAA |
| PIK3CA-3UTR-MUT-plasmid | AAAGATAACTGAGAAAATGAAAGCTCACTCTGGATTCCACACTGCACTGTTAATAACTCTCAGCAGGCAAAG**G**CCGATTGCATAGGAATTGCACAATCCATGAACAGCATTAGAATTTACAGCAAGAACAGAAATAAAATACTATATAATTTAAATAATGTAAACGCAAACAGAGAATGAAAATTTCTTATTTTTCCATTGCTGTTCAATTTATAGTTTGAAGTGGGTTTTTGACTGCTTGTTTAATGAAGAAAAATGCTTGGGGTGGAAGGG**T**CTCTTGAGATTTCACCAGAGACTTTTTCTTTTTAATAAATCAAACCTTTTGATGATTTGAGGTTTTATCTGCAGTTTTGGAAGCAGTCACAAATGAGACCGAATTAACACAATTTAGACTATCTTCCTGATTCCTTAAACCCCTTTACTGAAGTATACTCATGAATAATACTTTAAAATATGGGGGAATAGAAACCATGA**G**CTTTTTACCTTTTTAAACTATTTATCCATATCTCCAAAGTAGAACATTAAACCATTTTAAGATATGTCTCATTCCCAAGTAGTCAGAGCTCACTCTCCAACTTTATTAAATACTATTTGAGCACAGG**G**CACATTCTTAAACATTTTGAAAAACATTAACCCAAGATGTAGAGGCTACTGCTAGTCGTCATTCTAGAATCTGATATTTTACTCTGTATTTGAAATGAATAGAGTGAACATTGTATACTCTAGTAAAACAGCATCACTTTAAAAATATTCATTTATGAAATCTGTTACCTATAGTTGAAGTCTTGAGTAGTGAACAAGGG**G**CTCTAATACCAATACTCTTAATATCTGGCTATTTTAGATCCCTTAAAGGGCATAATTATTGGAAATTTAGGTATTTCACTAAAGCATGTATATAATATTGCATCCCAGCCTTTTCAATATCAGGGTTAAATTATAGGAAAACTCAGTAAAATGGTACAAATCTGAAAGTTTGATGGTAGAAACTGAAGATTTAACAGAGA**T**CTGTGTTTTACCCGAGTGCCAAAAATGCTGTGAGCCTCCTTGCACAAAATTTATACCACTTTTGCATTTTTATCTATCAGTCCAGATAGTTGTCTCCCCTGCACAAACCACATGGCCGATTTCACCATTTACATTTATTTTCAAAAGTTACTACAACCAAATTAATTCTATTAGAAGAAATGTAGACAAATTCTATAAAG**G**CTATAGATTGTGACCTAAGAAAGAAATGAGGCAAAGAACCAAACATTGAATTAAATGCTACATGGGTGACTAAGATCTGTTTCAAGTCAGTGATAATATAATTTGGGGCATGTCTTTAAGAGAAGGCTGAAAGTTGTGAGAGTATATTGTATACCGTAAGAGAATCAACTCTTCATCATGGATGGGATTGTGAAGGCTGA**G**CTATAAAATTCAGCATTGACAGCATCCTCAATTAATAATTCTTGGTGACAGAATAATACAGCTGGGCTGTTTTTTAAAATATAAACAATACCATTTTTAA |
| AKT1-3UTR-WT-plasmid | CTGACCAAGATGACAGCATGGAGTGTGTGGACAGCGAGCGCAGGCCCCACTTCCCCCAGTTCTCCTACTCGGCCAGCGGCACGGCCTGAGGCGGCGGTGG**A**CTGCGCTGGACGATAGCTTGGAGGGATGGAGAGGCGGCCTCGTGCCATGATCTGTATTTAATGGTTTTTATTTCTCGGGTGCATTTGAGAGAAGCCACGCTGTCCTCTCGAGCCCAGATGGAAAGACGTTTTTGTGCTGTGGGCAGCACCCTCCCCCGCAGCGGGGTAGGGAAGAAAACTATCCTGCGGGTTTTAATTTATTTCATCCAGTTTGTTCTCCGGGTGTGGCCTCAGCCCTCAGAACAATCCGATTCACGTAGGGAAATGTTAAGG**A**CTTCTGCAGCTATGCGCAATGTGGCATTGGGGGGCCGGGCAGGTCCTGCCCATGTGTCCCCTCACTCTGTCAGCCAGCCGCCCTGGGCTGTCTGTCACCA |
| AKT1-3UTR-MUT-plasmid | CTGACCAAGATGACAGCATGGAGTGTGTGGACAGCGAGCGCAGGCCCCACTTCCCCCAGTTCTCCTACTCGGCCAGCGGCACGGCCTGAGGCGGCGGTGG**G**CTGCGCTGGACGATAGCTTGGAGGGATGGAGAGGCGGCCTCGTGCCATGATCTGTATTTAATGGTTTTTATTTCTCGGGTGCATTTGAGAGAAGCCACGCTGTCCTCTCGAGCCCAGATGGAAAGACGTTTTTGTGCTGTGGGCAGCACCCTCCCCCGCAGCGGGGTAGGGAAGAAAACTATCCTGCGGGTTTTAATTTATTTCATCCAGTTTGTTCTCCGGGTGTGGCCTCAGCCCTCAGAACAATCCGATTCACGTAGGGAAATGTTAAGG**G**CTTCTGCAGCTATGCGCAATGTGGCATTGGGGGGCCGGGCAGGTCCTGCCCATGTGTCCCCTCACTCTGTCAGCCAGCCGCCCTGGGCTGTCTGTCACCA |

Supplementary Table 6. Demographic and clinical characteristics of participants in the

nested case-control study

| Variable | T2D  (n=100) | Control  (n=100) | *P* |
| --- | --- | --- | --- |
| Age (years) | 52.43 ± 6.91 | 52.43 ± 6.91 | — |
| Gender (male/female) | 54/46 | 54/46 | — |
| BMI (kg/m^2^) | 27.43 ± 3.91 | 26.11 ± 2.86 | 0.007 |
| SBP (mmHg) | 127.35 ± 14.42 | 123.04 ± 13.05 | 0.028 |
| DBP (mmHg) | 76.23 ± 10.62 | 73.44 ± 9.27 | 0.049 |
| TC (mmol/l) | 4.84 ± 0.86 | 4.62 ± 0.78 | 0.068 |
| TG (mmol/l) | 1.89 ± 1.18 | 1.52 ± 0.74 | 0.008 |
| LDLC (mmol/l) | 3.06 ± 0.86 | 2.85 ± 0.68 | 0.050 |
| HDLC (mmol/l) | 1.46 ± 0.36 | 1.53 ± 0.40 | 0.259 |
| FPG (mmol/l) | 5.33 ± 0.38 | 5.18 ± 0.42 | 0.313 |
| HbA1c (%) | 5.54 ± 0.29 | 5.47 ± 0.33 | 0.171 |
| Smoking (n) | 16 | 12 | 0.415^*^ |
| Alcohol use (n) | 17 | 16 | 0.894^*^ |
| Physical activity (n) | 74 | 80 | 0.313^*^ |

BMI: body mass index, SBP: systolic blood pressure, DBP: diastolic blood pressure, TC: total cholesterol, TG: triglyceride, LDLC: low-density lipoprotein cholesterol, HDLC: high-density lipoprotein cholesterol, FPG: fast plasma glucose, T2D: type 2 diabetes.

^*^ χ^2^ value.
